# The impact of learn, exercise, and game structured curriculum model on physical activity in children aged 3–6 years

**DOI:** 10.3389/fpsyg.2026.1881320

**Published:** 2026-07-20

**Authors:** Zuozheng Shi, Xulin Yang, Xi Long, Yongshun Li

**Affiliations:** 1 Early Childhood Sports and Health Research Centre at Chongqing Preschool Education College, Chongqing, China; 2 Nanchong Vocational College of Culture and Tourism, Nanchong, Sichuan, China

**Keywords:** early childhood physical education programs, LEG curriculum model, physical activity, preschoolers, structured curriculum

## Abstract

**Background:**

To investigate the impact of the LEG structured curriculum model on physical activity among children aged 3–6 years, thereby enhancing their levels of MVPA, TPA, and reducing SB.

**Methods:**

This study employed a quasi-experimental design with a repeated-measurements approach to implement a physical activity intervention for preschool children, with the aim of assessing their outdoor physical activity (OPA) and structured physical activity (SPA). Using a combination of random cluster sampling and stratified sampling, 180 preschool children aged 3–6 were selected as study participants. An Actigraph GT3X accelerometer was used to measure the children’s physical activity levels in order to evaluate the effectiveness of the program, and statistical analysis of the experimental data was conducted using an independent samples *t*-test and repeated measures analysis of variance.

**Results:**

In structured physical activities, both MVPA and TPA levels significantly increased among 3–6-year-old children in the experimental group. Among the 3–4-year-old children in the experimental group, MVPA increased by 68.5% at 6 weeks compared to baseline (8.14 vs. 4.83 min), but this increase had declined to 39.1% by 12 weeks. TPA also increased by 39% at 6 weeks (14.56 vs. 10.47 min), but by 12 weeks, the increase was only 7.6%, and exhibited a pattern of easy to initiate but difficult to sustain. For 4–5-year-old children in the experimental group, MVPA increased by 139.7% from week 0 to week 6 (12.78 vs. 5.33 min) and by an additional 30.1% by week 12. TPA increased by 84.5% at week 6 (21.14 vs. 11.46 min) and remained at a similar level at week 12, showed the most optimal intervention outcomes, with a linear increase. For 5–6-year-olds in the experimental group, MVPA increased by only 13.1% from baseline to week 6 (3.98 vs. 3.52 min), but surged by 251% by week 12 compared to week 6 (13.93 min). TPA also saw a significant increase by week 12 (a 140% increase compared to week 6), and influenced by factors such as class size and the children’s desire for autonomy.

**Conclusion:**

The LEG structured curriculum model is most effective and suitable for improving sedentary behavior and increasing physical activity levels among 4–5-year-old children. It also yields significant results for 3–4-year-olds and 5–6-year-olds; but the intervention strategies still need to be further optimized to enhance their sustainability and overall effectiveness.

## 1 Introduction

Preschool institutions play a vital role in promoting physical activity (PA) among children under 5 years old. However, in reality, these children spend the majority of their time being sedentary behavior (SB, 50–94%), with only a small portion engaged in light physical activity (LPA, 5–27%) or moderate-intensity physical activity (MPA, 1–17%) (Nicole, 2023). Insufficient physical activity in children may increase the risk of developing heart disease, hypertension, and obesity-related conditions in adulthood (Tucker, 2008), indicating that inadequate childhood physical activity has become a global public health concern. Currently, two-thirds of children worldwide do not meet the recommended levels of PA (Aubert and Barnes, 2018), making it imperative to increase children’s PA levels. PA is key to promoting sustained engagement in activities (Whitehead, 2013). MVPA during early childhood is associated with motor development (Guillermo et al., 2017; Kuzik et al., 2017), it significantly enhances fundamental motor skills (FMS) (Nilsen et al., 2020) and physical fitness (Barnett et al., 2016; Stodden et al., 2008). These studies indicate that engaging in MVPA during early childhood is more beneficial for children’s physical health development.

Research indicates that among preschool children’s activities, organized activities are more effective than free play in generating high levels of physical engagement (Palmer et al., 2017; Tortella et al., 2019). Both organized indoor and outdoor physical activity environments significantly influence preschoolers’ PA behaviors and motor development (Niemistö et al., 2019; Terrón-Pérez et al., 2021). Furthermore, organized physical activities predominate in both physical education classes and extracurricular activities. Preschooler’s MVPA primarily occurs in structured settings through fixed activity schedules and free play periods. This relatively stable form of activity is not easily replaced by other types of activities (Shi and Liu, 2024). However, current structured curriculum models have yet to improve the insufficient PA levels among preschool children.

A study examining the relationship between MVPA duration in elementary school physical education classes and teachers’ instructional behaviors revealed that when teachers engaged in positive feedback, movement demonstration, and instructional guidance behaviors, students’ MVPA duration was significantly higher compared to other instructional behaviors (Chow et al., 2008; Martin and Kulinna, 2005). Chow et al. (2008) suggest that physical drills, skill practice, games, and free play contribute to enhancing students’ PA levels. Among classroom factors, skill practice and game time represent modifiable variables that positively influence students’ percentage of MVPA during physical education classes (Zhou, 2020). These findings provide a basis for developing new structured curriculum models; however, current research has not yet clearly identified which curriculum model is effective in increasing physical activity levels among young children.

The low-intensity, self-selected, and unstructured outdoor activities implemented in kindergartens may lead to insufficient exercise effects, potentially contributing to delayed development of FMS. There is an urgent need for moderate-to-high-intensity, structured physical activities employing a “teach, practice diligently, compete regularly” approach to facilitate continuous practice and feedback. This enables effective control over movements, thereby sustaining active participation in physical activities across diverse environments (Ning et al., 2022). Specifically, NASPE states that structured movement and physical activity opportunities should encourage the development of FMS as well as utilize all the large muscle groups (National Association for Sport and Physical Education, 2010). A study on the CHAMP curriculum intervention noted that MVPA remains under-validated, recommending that preschools consider providing diverse opportunities for gross motor activities. These should include outdoor recess activities, structured physical education classes, as well as physical activities and movement breaks integrated into classroom instruction (Palmer et al., 2017). The physical activity support provided by structured programs relies heavily on ongoing external resources; once that external support is reduced or discontinued, the programs’ ability to promote physical activity may diminish accordingly (Lee et al., 2024). Currently, although researchers have proposed several recommendations regarding structured curricula, findings demonstrating the effectiveness of structured curricula in improving young children’s PA proficiency are rarely reported.

Research indicates that tasks involving high cognitive load can directly enhance students’ participation in physical education classes (Deng and Chen, 2023), this effect depends on whether individuals possess the necessary cognitive processing abilities, suggesting that structured curricula can have varying impacts on physical activity among children of different ages. Therefore, structured curriculum interventions must incorporate age-differentiated strategies: for 3–4-year-olds, curriculum content should focus primarily on mobility skills, supplemented by manipulative skills; for 4–5-year-olds, the focus should shift to manipulative skills, with stability skills gradually integrated; and for 5–6-year-olds, the emphasis should be on stability skills, with the comprehensive application of mobility, stability, and manipulative skills.

It is clear that there are significant differences in the implementation of structured physical education programs due to factors such as the environment, age, teaching practices, and instructional models; these differences may directly impact the effectiveness of such programs in promoting physical activity among children. Existing research tends to focus on achieving curriculum objectives through teacher training, task design, and gamified instruction, but it has not fully taken into account the phased and continuous nature of young children’s physical and mental development. There is a lack of systematic and holistic design of curriculum content, which may be the reason for the insufficient level of physical activity among young children.

Based on this, we designed the learn movement, exercise skill, and game activities (LEG) structured curriculum model. This curriculum model is based on the design philosophy of “learning through exploration, mastering through exercise, applying through game activities, and interacting at home and in the community.” It aims to enhance young children’s physical activity levels from multiple perspectives—at home, in the community, and in the classroom. The model has been proven to possess strong scientific validity and practical applicability in terms of curriculum objectives and content (Shi et al., 2025a), demonstrating significant effects on physical fitness development (Shi et al., 2025b). However, its impact on children’s PA remains unproven.

Due to young children’s limited ability to sustain attention, intervention sessions that are either too long or too short will not yield optimal benefits (Logan et al., 2012). According to research on intervention effectiveness indicates that sessions lasting 30–40 min, conducted at least twice weekly over a total duration of 8–16 weeks, maximize intervention outcomes (Logan et al., 2012; Donath et al., 2015). Therefore, this study examined the effects of a 12-week experimental intervention program comprising 24 sessions, each lasting at least 30 min, on outdoor physical activity (OPA) and structured physical activity (SPA) among preschool children aged 0–12 weeks. The OPA reflects young children’s physical activity levels during their entire 600-min day at preschool, while SPA reflects their physical activity levels during 40 min of structured classes. Measuring and analyzing SPA provides a clear indication of the effectiveness of the LEG structured curriculum model. This study hypothesizes that, at the conclusion of the intervention, the preschool children in the experimental group will demonstrate higher levels of SB, LPA, MPA, vigorous physical activity (VPA), MVPA, and total physical activity (TPA) compared to their pre-intervention levels, and that these levels will be higher than those of the control group. This will serve to evaluate the effectiveness of the LEG structured curriculum model in improving physical activity levels among children aged 3–6 years.

## 2 Materials and methods

### 2.1 Subjects

**FIGURE 1:**
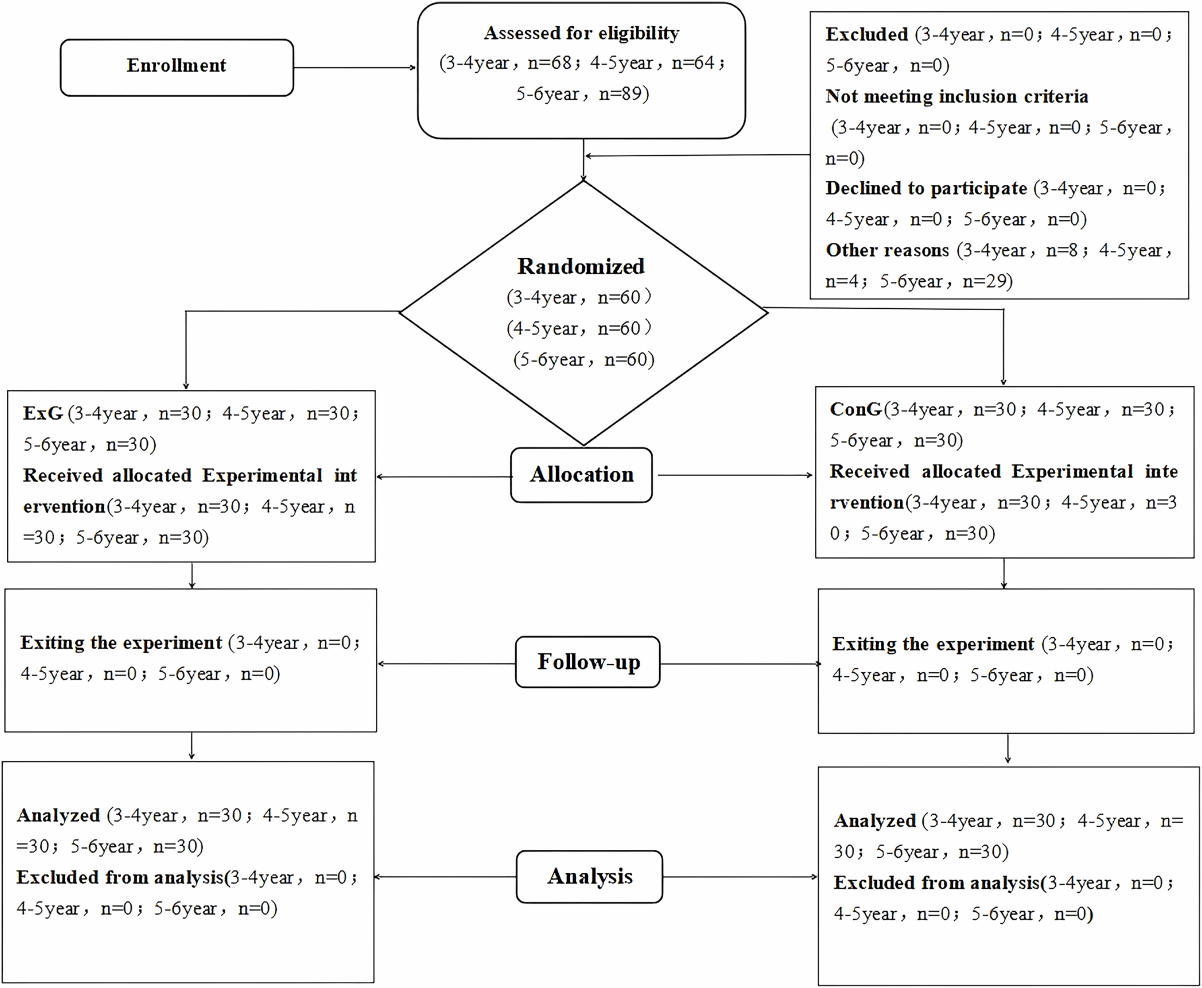
Project recruitment flowchart.

This project was conducted at a kindergarten located in an urban-rural fringe area of an economically underdeveloped region in western China. After obtaining informed consent from participants and their guardians, a total of 221 children aged 3–6 years were recruited. In this study, experimental and control groups were assigned to each age group through random assignment, with the randomization process conducted by members of the research team. Preschool teachers from each class selected a total of 180 children—30 from each of the six classes in batches—as official participants, based on pre-established inclusion and exclusion criteria. Eligibility criteria include: (1) no physical disabilities or medical conditions that require restricted activity. (2) Parents have signed an informed consent form. (3) Participants are not enrolled in specialized extracurricular sports programs. To minimize the impact of outliers on the evaluation of the intervention’s effectiveness, teachers first excluded children in each class whose physical activity levels fell within the top 10% (very active) and bottom 10% (very inactive) based on at least 1 week of daily observations, they then randomly selected a sufficient sample size in batches from the remaining children. The final group of preschoolers wearing Actigraph GT3X accelerometers participated in an intervention study conducted in a natural classroom setting. The randomization process process is shown in the Figure 1.

### 2.2 Experimental protocol

Young children should master fundamental motor skills encompassing mobility, manipulation, and stability (Higgs et al., 2008). These skills will be applied through movement sequencing to meet the regulatory demands of physical activity. Therefore, we designed eight mobility, manipulation, and stability movements tailored to the physical and mental developmental characteristics of preschool children aged 3–6. These movements were developed to assess the impact of the experimental curriculum on the PA levels of children in this age group. For details, please refer to course plans P1-3.

In the experiment, participants were required to complete a 12-week intervention program consisting of two sessions per week, each lasting at least 30 min, a total of at least 720 min of experimental intervention sessions, incorporating basic motor skills such as walking, running, jumping, and throwing, with clearly defined objectives. The experimental group strictly followed the LEG structured curriculum model and content design steps: (1) Movement Activation (10%); (2) Movement Exploration and Learning (20%); (3) Skill Reinforcement Practice (20%); (4) Game-based practice activities (40%); (5) Relaxation activities (10%), as detailed in Experimental course cases E1-3. The control group primarily utilized semi-structured and unstructured curricula, a form of physical activity that lacks predetermined, standardized learning objectives, a fixed procedure, and mandatory instruction, see C1-3 for examples of routine curriculum implementation.

To evaluate the actual effectiveness of the experimental curriculum, the experimental program was implemented after being trained and assessed by members of the Early Childhood Physical Education and Health Research Center at Chongqing Preschool Education College. Additionally, considering that differences in teaching styles among instructors may influence experimental outcomes, each experimental course must meet the following six evaluation Point. The implementation process of each course must be recorded and analyzed via video documentation, as detailed in Table 1 (Shi et al., 2025b).

**TABLE 1:** Assessment points for the experimental intervention program.

No	Evaluation point
1	Is the teaching content for basic motor skills in preschoolers clearly defined in the experimental curriculum?
2	Did the children participate in practicing basic motor skills during the experimental course?
3	Were gamified teaching activities implemented according to the established curriculum model and content in the experimental course to reinforce fundamental motor skills?
4	Are the teacher’s demonstration of actions, verbal instructions, and organization of activities in the experimental course appropriate?
5	Is the time allocation across the five segments of the experimental course motor activation, movement exploration and learning, skill reinforcement practice, game-based activities, and relaxation activities reasonable?
6	Is there a connection between the content of the experimental course and the five components: movement activation, action exploration learning, skill reinforcement practice, game-based activities, and relaxation activities?

### 2.3 Measurement and evaluation

The Actigraph GT3X accelerometer (Model GT3X; Actigraph LLC, Pensacola, FL, United States) was worn on the subject’s hip for 600 min daily to assess children’s OPA and SPA. Accelerometers were distributed and worn by preschool teachers upon children’s arrival at the kindergarten and collected uniformly upon departure. Experimental data were collected at 15-s intervals and processed and analyzed using Actilife 6.0 software Actigraph. Based on the equipment and computational models, the intensity thresholds defined are as follows (Butte et al., 2014), SB threshold [0, 819], LPA threshold [819, 3,907], MPA threshold [3,907, 6,111], VPA threshold [6,112, +∞), and recorded MPA and VPA as MVPA, LPA and MVPA as TPA. Accelerometers were checked before each test to ensure children’s SPA were recorded properly.

Baseline PA were assessed prior to the experimental intervention (0W). Mid-intervention PA were measured at week 6 of the experimental intervention (6W). Late-intervention PA were evaluated at week 12 of the experimental intervention (12W). The participants’ SPA data were extracted from the control group (regional play activities) and the experimental group (experimental intervention program) and used for analysis, with a data extraction time of 40 min. Since OPA encompasses SPA, and changes in SPA inevitably lead to changes in OPA, this study will focus on discussing and analyzing SPA.

### 2.4 Statistical analysis

Data were analyzed using SPSS 24.0. All data are presented as mean ± standard deviation (M ± SD). Baseline comparisons between groups were performed using an independent samples *t*-test. The intervention effect was evaluated using a 2 (group: experimental group, control group) × 3 (time: 0, 6, 12 weeks) repeated measures analysis of variance (ANOVA). The sphericity assumption was tested prior to analysis (Mauser’s test), if violated, the Greenhouse-Geisser correction was applied. The normality of the residuals was assessed using the Shapiro-Wilk test and Q-Q plots. *Post-hoc* pairwise comparisons and simple effects analysis were corrected for multiple comparisons using the Bonferroni method, and corrected *p*-values were reported. The overall significance level was set at α = 0.05 (two-tailed). Effect sizes were reported as partial η^2^ and their 95% confidence intervals.

## 3 Results

### 3.1 Basic information and characteristics of participants

A total of 180 preschool children aged 3–6 years completed OPA and SPA measurements and assessments from week 0 to week 12 in this project, with boys and girls each accounting for 50%, as shown in Table 2.

**TABLE 2:** Basic characteristics of subjects in the experimental and control groups.

Age	ExG				ConG			
	n	Height (cm)	Weight (kg)	BMI (kg/m^2^)	n	Height (cm)	Weight (kg)	BMI (kg/m^2^)
3–4 Years	30	102.05	17.40	16.67	30	102.41	17.47	16.62
4–5 Years	30	110.12	20.06	16.50	30	110.92	19.87	16.13
5–6 Years	30	115.75	21.38	15.96	30	117.47	22.48	16.23
Total	90	109.31	19.61	16.38	90	110.27	19.94	16.33

### 3.2 Comparison of OPA and SPA at week 0 between the experimental and control groups of 3–6-year-old participants

Table 3 presents the results of the independent samples *t*-test comparing the OPA and SPA durations at Week 0 between the experimental and control groups. The findings indicate that the differences in OPA and SPA at week 0 between the experimental and control groups were not statistically significant (*p* > 0.05).

**TABLE 3:** Comparison of OPA and SPA at week 0 between the experimental and control groups of 3–6-year-old participants.

Test item		3–4 years			4–5 years			5–6 years		
		ExG (*n* = 30)	ConG (*n* = 30)	*P*	ExG (*n* = 30)	ConG (*n* = 30)	*P*	ExG (*n* = 30)	ConG (*n* = 30)	*P*
OPA										
	SB	547.28 ± 3.59	547.63 ± 17.95	0.915	508.75 ± 8.28	508.93 ± 7.94	0.934	506.25 ± 12.60	507.18 ± 8.22	0.737
	LPA	32.27 ± 1.97	31.46 ± 9.61	0.653	44.75 ± 1.90	44.72 ± 1.64	0.942	48.82 ± 6.67	47.24 ± 3.38	0.253
	MPA	16.33 ± 1.23	16.19 ± 7.42	0.923	30.73 ± 3.49	30.84 ± 3.52	0.905	27.79 ± 5.60	26.17 ± 2.41	0.150
	VPA	4.95 ± 0.62	4.72 ± 1.98	0.540	15.70 ± 4.45	15.53 ± 4.26	0.877	17.14 ± 4.33	16.46 ± 0.94	0.401
	MVPA	21.28 ± 1.79	20.91 ± 9.13	0.830	46.43 ± 7.53	46.37 ± 7.50	0.973	44.93 ± 6.68	42.63 ± 2.49	0.081
	TPA	52.71 ± 3.58	52.37 ± 17.95	0.919	91.18 ± 8.19	91.09 ± 7.98	0.965	93.75 ± 12.60	92.83 ± 8.22	0.737
SPA										
	SB	29.44 ± 1.80	29.44 ± 1.80	0.900	28.54 ± 3.87	28.99 ± 2.93	0.614	31.96 ± 2.23	32.73 ± 1.98	0.163
	LPA	5.63 ± 0.82	5.59 ± 0.72	0.835	6.13 ± 1.88	6.04 ± 1.74	0.845	4.53 ± 1.51	4.43 ± 1.35	0.798
	MPA	3.87 ± 1.33	3.80 ± 1.00	0.827	3.50 ± 1.40	3.42 ± 0.97	0.790	2.43 ± 0.96	2.43 ± 0.82	1.000
	VPA	0.97 ± 0.29	1.01 ± 0.29	0.581	1.83 ± 1.61	1.48 ± 0.23	0.243	1.08 ± 0.53	1.10 ± 0.49	0.900
	MVPA	4.83 ± 1.55	5.00 ± 1.20	0.643	5.33 ± 2.63	4.89 ± 1.16	0.413	3.52 ± 0.91	3.53 ± 0.84	0.942
	TPA	10.47 ± 2.27	10.56 ± 1.80	0.863	11.46 ± 3.87	10.97 ± 2.89	0.579	8.04 ± 2.23	8.33 ± 2.19	0.611

#### 3.3 Comparison of SPA in 3–4-year-old subjects in the experimental and control groups from 0 to 12 weeks

Table 4 presents the results of our repeated-measures analysis of variance [2 (group: experimental group, control group) × 3 (time: 0, 6, 12 weeks)] for the SPA in 3–4-year-old participants. The Mauchly sphericity test indicated that SB, LPA, MPA, VPA, MVPA, and TPA violated the sphericity assumption (*p* < 0.05); Greenhouse-Geisser correction was applied (ε = 0.813, 0.884, 0.831, 0.732, 0.760, and 0.808, respectively). The Shapiro-Wilk test and Q-Q plots of the standardized residuals indicated that the residuals were generally normally distributed. All *post-hoc* pairwise comparisons and simple effects analyses were adjusted using the Bonferroni correction, and the corrected *p*-values are reported.

**TABLE 4:** Comparison of SPA in 3–4-year-old subjects in the experimental and control groups from 0 to 12 weeks.

Test item	ExG (*n* = 30)			ConG (*n* = 30)			Group		Time		Interaction	
	0 w	6 w	12 w	0 w	6 w	12 w	Main effect *F*-value (ES)	*P*	Main effect *F*-value (ES)	*P*	Interaction *F*-value (ES)	*P*
SB	29.44 ± 1.80	25.44 ± 4.99	28.80 ± 1.69	29.44 ± 1.80	31.63 ± 1.84	30.25 ± 2.92	25.224 (0.303)	0.000**	2.971 (0.049)	0.067	25.144 (0.302)	0.000**
LPA	5.63 ± 0.82	6.42 ± 2.18	4.55 ± 1.02	5.59 ± 0.72	3.81 ± 0.94	4.16 ± 0.92	22.928 (0.283)	0.000**	20.499 (0.261)	0.000**	24.771 (0.299)	0.000**
MPA	3.87 ± 1.33	5.94 ± 2.28	3.81 ± 0.93	3.80 ± 1.00	3.00 ± 0.80	3.88 ± 1.48	12.099 (0.173)	0.000**	4.696 (0.075)	0.016*	33.019 (0.363)	0.000**
VPA	0.97 ± 0.29	2.20 ± 1.11	2.91 ± 1.12	1.01 ± 0.29	1.42 ± 0.43	1.71 ± 0.91	33.484 (0.366)	0.000**	43.071 (0.426)	0.000**	9.781 (0.144)	0.000**
MVPA	4.83 ± 1.55	8.14 ± 3.19	6.72 ± 1.44	5.00 ± 1.20	4.42 ± 1.11	5.59 ± 2.14	22.867 (0.283)	0.000**	10.493 (0.153)	0.000**	19.271 (0.249)	0.000**
TPA	10.47 ± 2.27	14.56 ± 4.99	11.27 ± 1.69	10.56 ± 1.80	8.36 ± 1.84	9.75 ± 2.92	26.244 (0.312)	0.000**	2.777 (0.046)	0.078	24.768 (0.299)	0.000**

*p< 0.05, **p< 0.01.

The results showed that the main effect of the experimental group SB was significant [*F* = 25.224, *p* < 0.001, partial η^2^ = 0.303, 95% CI (0.16, 0.44)]; the main effect of time was not significant [*F* = 2.971, *p* = 0.067, ES = 0.049, 95% CI (0.00, 0.14)], and the interaction between time and group was significant [*F* = 25.144, *p* < 0.001, partial η^2^ = 0.302, 95% CI (0.17, 0.43)]. Further simple effects analysis showed that the experimental group’s t1 score was significantly higher than t2 (*p* < 0.001) and t3 was significantly higher than t2 (*p* < 0.001), but there was no significant difference between t3 and t1 (*p* = 0.621); In the control group, t2 was significantly higher than t1 (*p* < 0.001), but there were no significant differences between t3 and either t1 or t2 (*p* > 0.05).

The main effect of LPA in the experimental group was significant [*F* = 22.928, *p* < 0.001, partial η^2^ = 0.283, 95% CI (0.14, 0.42)]; The main effect of time was significant [*F* = 20.499, *p* < 0.001, partial η^2^ = 0.261, 95% CI (0.13, 0.39)]; The interaction between time and group was significant [*F* = 24.771, *p* < 0.001, partial η^2^ = 0.299, 95% CI (0.17, 0.43)]. Further simple effects analysis showed that in the experimental group, t1 was significantly lower than t2 (*p* = 0.023), and t3 was significantly lower than both t1 and t2 (*p* < 0.001); In the control group, both t2 and t3 were significantly lower than t1 (*p* < 0.001), but there was no significant difference between t3 and t2 (*p* = 0.825).

The main effect of MPA in the experimental group was significant (*F* = 12.099, *p* < 0.001, partial η^2^ = 0.173, 95% CI (0.05, 0.31)]; the main effect of time was significant [*F* = 4.696, *p* = 0.016, ES = 0.075, 95% CI (0.00, 0.18)]; the interaction between time and group was significant [*F* = 33.019, *p* < 0.001, partial η^2^ = 0.363, 95% CI (0.22, 0.48)]. Further *post-hoc* analyses revealed that t2 in the experimental group was significantly higher than t1 and t3 (*p* < 0.001), while there was no significant difference between t3 and t1 (*p* = 1.000); In the control group, t2 was significantly lower than t1 (*p* < 0.001), and there were no significant differences between t3 and either t1 or t2 (*p* > 0.05).

The main effect of VPA in the experimental group was significant [*F* = 33.48, *p* < 0.001, partial η^2^ = 0.366, 95% CI (0.22, 0.49)]; the main effect of time was significant [*F* = 43.071, *p* < 0.001, partial η^2^ = 0.426]; the interaction between time and group was significant [*F* = 9.781, *p* < 0.001, partial η^2^ = 0.144]. Further simple effects analysis showed that in the experimental group, t2 and t3 were both significantly higher than t1 (*p* < 0.001), and t3 was significantly higher than t2 (*p* = 0.036); In the control group, t2 and t3 were both significantly higher than t1 (*p* = 0.020, *p* = 0.002), but there was no significant difference between t3 and t2 (*p* = 0.776).

The main effect of MVPA in the experimental group was significant [*F* = 22.867, *p* < 0.001, partial η^2^ = 0.283, 95% CI (0.14, 0.42)]; the main effect of time was significant [*F* = 10.493, *p* < 0.001, partial η^2^ = 0.153, 95% CI (0.04, 0.27)]. The interaction between time and group was significant [*F* = 19.271, *p* < 0.001, partial η^2^ = 0.249, 95% CI (0.11, 0.38)]. Further simple effects analysis showed that in the experimental group, t2 and t3 were both significantly higher than t1 (*p* < 0.001), and t3 was significantly lower than t2 (*p* = 0.044); There were no significant differences between the various time points within the control group (*p* > 0.05).

The main effect of TPA in the experimental group was significant [*F* = 26.244, *p* < 0.001, partial η^2^ = 0.312, 95% CI (0.16, 0.45)]; the main effect of time was significant [*F* = 2.777, *p* = 0.078, ES = 0.046, 95% CI (0.00, 0.13)], and the interaction between time and group was significant [*F* = 24.768, *p* < 0.001, partial η^2^ = 0.299, 95% CI (0.16, 0.42)]. Further simple effects analysis showed that the experimental group’s t2 score was significantly higher than t1 and t3 (*p* < 0.001), while there was no significant difference between t3 and t1 (*p* = 0.517); In the control group, t2 was significantly lower than t1 (*p* < 0.001), t3 was significantly higher than t2 (*p* = 0.088), but there was no significant difference between t3 and t1 (*p* > 0.05).

### 3.4 Comparison of SPA in 4–5-year-old subjects in the experimental and control groups from week 0 to week 12

Table 5 presents the results of our repeated-measures analysis of variance [2 (group: experimental group, control group) × 3 (time: 0, 6, 12 weeks)] for the SPA in 4–5-year-old participants. The Mauchly sphericity test indicated that SB, LPA, MPA, MVPA, and TPA violated the sphericity assumption (*p* < 0.05); Greenhouse-Geisser correction was applied (ε = 0.774, 0.741, 0.776, 0.907, and 0.774, respectively). VPA met the sphericity assumption (*p* > 0.05). The Shapiro-Wilk test and Q-Q plots of standardized residuals indicated that the residuals were generally normally distributed. All *post-hoc* pairwise comparisons and simple effects analyses were adjusted using the Bonferroni correction, and corrected *p*-values are reported.

**TABLE 5:** Comparison of SPA in 4–5-year-old subjects in the experimental and control groups from week 0 to week 12.

Test item	ExG (*n* = 30)			ConG (*n* = 30)			Group		Time		Interaction	
	0 w	6 w	12 w	0 w	6 w	12 w	Main effect *F*-value (ES)	*P*	Main effect *F*-value (ES)	*P*	Interaction *F*-value (ES)	*P*
SB	28.54 ± 3.87	18.86 ± 3.51	19.18 ± 1.23	28.99 ± 2.93	29.55 ± 3.25	28.05 ± 2.78	191.603 (0.768)	0.000**	54.831 (0.486)	0.000**	51.475 (0.470)	0.000**
LPA	6.13 ± 1.88	8.36 ± 1.18	4.19 ± 0.70	6.04 ± 1.74	4.96 ± 1.33	5.43 ± 1.31	14.613 (0.201)	0.000**	25.528 (0.306)	0.000**	40.312 (0.410)	0.000**
MPA	3.50 ± 1.40	7.93 ± 1.39	5.97 ± 0.90	3.42 ± 0.97	4.00 ± 1.51	4.43 ± 1.37	82.345 (0.587)	0.000**	65.150 (0.529)	0.000**	37.319 (0.392)	0.000**
VPA	1.83 ± 1.61	4.86 ± 1.69	10.66 ± 1.43	1.48 ± 0.23	1.49 ± 0.87	2.10 ± 1.22	442.288 (0.884)	0.000**	220.045 (0.791)	0.000**	162.668 (0.737)	0.000**
MVPA	5.33 ± 2.63	12.78 ± 2.82	16.63 ± 1.05	4.89 ± 1.16	5.48 ± 2.10	6.53 ± 1.63	322.809 (0.848)	0.000**	176.016 (0.752)	0.000**	102.104 (0.638)	0.000**
TPA	11.46 ± 3.87	21.14 ± 3.51	20.82 ± 1.23	10.97 ± 2.89	10.45 ± 3.25	11.95 ± 2.78	19.700 (0.769)	0.000**	55.576 (0.489)	0.000**	51.343 (0.470)	0.000**

*p < 0.05, **p < 0.01.

The results showed that the main effect of SB in experimental group was significant [*F* = 191.603, *p* < 0.001, partial η^2^ = 0.768, 95% CI (0.68, 0.83)]; the main effect of Time was significant [*F* = 54.831, *p* < 0.001, partial η^2^ = 0.486, 95% CI (0.36, 0.59)]; The interaction between time and group was significant [*F* = 51.475, *p* < 0.001, partial η^2^ = 0.470, 95% CI [0.34, 0.57)]. Further simple effects analysis showed that t2 and t3 in the experimental group were both significantly lower than t1 (*p* < 0.001), but there was no significant difference between t2 and t3 (*p* = 1.000); In the control group, t3 was significantly lower than t2 (*p* = 0.047), but there were no significant differences between t1 and t2 or between t1 and t3 (*p* > 0.05).

The main effect of LPA in the experimental group was significant [*F* = 14.613, *p* < 0.001, partial η^2^ = 0.201, 95% CI (0.07, 0.34)]; the main effect of time was significant [*F* = 25.528, *p* < 0.001, partial η^2^ = 0.306, 95% CI (0.17, 0.43)]; the interaction between time and group was significant [*F* = 40.312, *p* < 0.001, partial η^2^ = 0.410, 95% CI (0.26, 0.53)]; further simple effects analysis showed that in the experimental group, t1 was significantly lower than t2 (*p* < 0.001), and t3 was significantly lower than both t1 and t2 (*p* < 0.001); in the control group, there were no significant differences between any of the time points (*p* > 0.05).

The main effect of MPA in the experimental group was significant [*F* = 82.345, *p* < 0.001, partial η^2^ = 0.587, 95% CI (0.47,0.68)]. The main effect of time was significant (*F* = 65.150, *p* < 0.001, partial η^2^ = 0.529, 95% CI (0.41, 0.62)]. The interaction between time and group was significant [*F* = 37.319, *p* < 0.001, partial η^2^ = 0.392, 95% CI (0.25, 0.51)]. Further simple effects analysis showed that in the experimental group, t2 and t3 were both significantly higher than t1 (*p* < 0.001), and t3 was significantly lower than t2 (*p* < 0.001); In the control group, t3 was significantly higher than t1 (*p* = 0.011), but there were no significant differences between t1 and t2, or between t2 and t3 (*p* > 0.05).

The main effect of VPA in the experimental group was significant [*F* = 442.288, *p* < 0.001, partial η^2^ = 0.884, 95% CI (0.84,0.91)]; The main effect of time was significant [*F* = 220.045, *p* < 0.001, partial η^2^ = 0.791, 95% CI (0.74, 0.83)]; The interaction between time and group was significant [*F* = 162.668, *p* < 0.001, partial η^2^ = 0.737, 95% CI (0.67, 0.79)]. Further simple effects analysis showed that the experimental group’s scores at t2 and t3 were both significantly higher than at t1 (*p* < 0.001), and t3 was significantly higher than t2 (*p* = 0.000); No significant differences were found in any pairwise comparisons across all time points in the control group (*p* > 0.05).

The main effect of MVPA in the experimental group was significant [*F* = 322.809, *p* < 0.001, partial η^2^ = 0.848, 95% CI (0.79, 0.89)]; The main effect of time was significant [*F* = 176.016, *p* < 0.001, partial η^2^ = 0.752, 95% CI (0.68, 0.81)]; The interaction between time and group was significant [*F* = 102.104, *p* < 0.001, partial η^2^ = 0.638, 95% CI (0.54, 0.71)]. Further simple effects analysis showed that the experimental group’s scores at t2 and t3 were both significantly higher than at t1 (*p* < 0.001), and t3 was significantly higher than t2 (*p* = 0.000); In the control group, t3 was significantly higher than both t1 and t2 (*p* = 0.001, *p* = 0.042), but there was no significant difference between t1 and t2 (*p* = 1.000).

The main effect of TPA in the experimental group was significant [*F* = 19.700, *p* < 0.001, partial η^2^ = 0.769, 95% CI (0.68, 0.83)]; The main effect of time was significant [*F* = 55.576, *p* < 0.001, partial η^2^ = 0.489, 95% CI (0.37, 0.59)]; The interaction between time and group was significant [*F* = 51.343, *p* < 0.001, partial η^2^ = 0.470, 95% CI (0.34, 0.57)]. Further simple effects analysis showed that t2 and t3 in the experimental group were both significantly higher than t1 (*p* < 0.001), but there was no significant difference between t2 and t3 (*p* = 1.000); In the control group, t3 was significantly higher than t2 (*p* = 0.046), but there were no significant differences between t1 and t2 or between t1 and t3 (*p* > 0.05).

### 3.5 Comparison of SPA in 5–6-year-old subjects in the experimental and control groups from week 0 to week 12

Table 6 presents the results of the repeated-measures ANOVA (Groups: Experimental Group, Control Group; Time: week 0, week 6, week 12) conducted on the SPA scores of 5–6-year-old participants. The Munchly sphericity test indicated that LPA and VPA violated the sphericity assumption (*p* < 0.05); Greenhouse-Geisser correction was applied (ε = 0.869 and 0.799, respectively); SB, MPA, MVPA, and TPA met the sphericity assumption (*p* > 0.05). The Shapiro-Wilk test and Q-Q plots of the standardized residuals indicated that the residuals were generally normally distributed. All *post-hoc* pairwise comparisons and simple effects analyses were corrected using the Bonferroni correction, and corrected *p*-values are reported.

**TABLE 6:** Comparison of SPA in 5–6-year-old subjects in the experimental and control groups from week 0 to week 12.

Test item	ExG (*n* = 30)			ConG (*n* = 30)			Group		Time		Interaction	
	0 w	6 w	12 w	0 w	6 w	12 w	Main effect *F*-value (ES)	*P*	Main effect *F*-value (ES)	*P*	Interaction *F*-value (ES)	*P*
SB	31.96 ± 2.23	31.91 ± 3.66	20.55 ± 3.18	32.73 ± 1.98	29.41 ± 5.29	31.36 ± 2.07	25.358 (0.304)	0.000**	81.760 (0.585)	0.000**	89.681 (0.607)	0.000**
LPA	4.53 ± 1.51	4.12 ± 1.77	5.53 ± 1.18	4.43 ± 1.35	5.98 ± 2.96	5.02 ± 0.79	1.939 (0.032)	0.169	4.118 (0.066)	0.024*	9.885 (0.146)	0.000**
MPA	2.43 ± 0.96	2.83 ± 1.22	6.18 ± 1.42	2.43 ± 0.82	3.51 ± 2.11	2.98 ± 1.25	9.769 (0.144)	0.003**	61.057 (0.513)	0.000**	54.896 (0.486)	0.000**
VPA	1.08 ± 0.53	1.15 ± 1.06	7.75 ± 2.13	1.10 ± 0.49	1.10 ± 0.61	0.64 ± 0.33	207.547 (0.782)	0.000**	181.060 (0.757)	0.000**	238.802 (0.805)	0.000**
MVPA	3.52 ± 0.91	3.98 ± 2.10	13.93 ± 2.92	3.53 ± 0.84	4.61 ± 2.58	3.63 ± 1.47	81.692 (0.585)	0.000**	162.190 (0.737)	0.000**	190.215 (0.766)	0.000**
TPA	8.04 ± 2.23	8.09 ± 3.66	19.45 ± 3.18	8.33 ± 2.19	10.59 ± 5.29	8.64 ± 2.07	18.810 (0.245)	0.000**	73.593 (0.559)	0.000**	97.179 (0.626)	0.000**

*p < 0.05, **p< 0.01.

The results showed that the main effect of SB was significant [*F* = 25.358, *p* < 0.001, partial η^2^ = 0.304, 95% CI (0.15, 0.45)]; the main effect of Time was significant [*F* = 81.760, *p* < 0.001, partial η^2^ = 0.585, 95% CI (0.49,0.66)]; the interaction between Time and Group was significant [*F* = 89.681, *p* < 0.001, partial η^2^ = 0.607, 95% CI (0.51, 0.68)]. Further simple effects analysis showed that t3 in the experimental group was significantly lower than t1 and t2 (*p* < 0.001), but there was no significant difference between t1 and t2 (*p* = 1.000); in the control group, t2 was significantly lower than t1 (*p* < 0.001), t3 was significantly higher than t2 (*p* = 0.041), and there was no significant difference between t3 and t1 (*p* = 0.119).

In the experimental group, the main effect of LPA was not significant [*F* = 1.939, *p* = 0.169, partial η^2^ = 0.03, 95% CI (0.00, 0.13)], while the main effect of time was significant [*F* = 4.118, *p* = 0.024, ES = 0.066, 95% CI (0.00, 0.16)]; The interaction between time and group was significant [*F* = 9.885, *p* < 0.001, partial η^2^ = 0.146, 95% CI (0.05, 0.25)]. Further simple effects analysis showed that t3 in the experimental group was significantly higher than t1 and t2 (*p* = 0.007), but there was no significant difference between t1 and t2 (*p* = 1.000); In the control group, t2 was significantly higher than t1 (*p* = 0.003), and there were no significant differences between t3 and either t1 or t2 (*p* = 0.316, *p* = 0.095).

The main effect of MPA in the experimental group was significant [*F* = 9.769, *p* = 0.003, partial η^2^ = 0.144, 95% CI (0.03, 0.28)]; The main effect of time was significant [*F* = 61.057, *p* < 0.001, partial η^2^ = 0.513, 95% CI (0.39, 0.61)]; The interaction between time and group was significant [*F* = 54.896, *p* < 0.001, partial η^2^ = 0.486, 95% CI (0.36, 0.59)]. Further *post-hoc* analyses revealed that t3 in the experimental group was significantly higher than t1 and t2 (*p* < 0.001), but there was no significant difference between t1 and t2 (*p* = 0.438); In the control group, t2 was significantly higher than t1 (*p* = 0.000), and there were no significant differences between t3 and t1 or t2 (*p* = 0.148, *p* = 0.082).

The main effect of VPA in the experimental group was significant [*F* = 207.547, *p* < 0.001, partial η^2^ = 0.782, 95% CI (0.70, 0.84)]; The main effect of time was significant [*F* = 181.060, *p* < 0.001, partial η^2^ = 0.757, 95% CI (0.68, 0.81)]; The interaction between time and group was significant [*F* = 238.802, *p* < 0.001, partial η^2^ = 0.805, 95% CI (0.75, 0.85)]. Further simple effects analysis showed that the experimental group’s t3 score was significantly higher than t1 and t2 (*p* < 0.001), but there was no significant difference between t1 and t2 (*p* = 1.000); In the control group, there were no significant differences between t1, t2, and t3 (*p* > 0.05).

The main effect of MVPA in the experimental group was significant [*F* = 81.692, *p* < 0.001, partial η^2^ = 0.585, 95% CI (0.46,0.68)]; The main effect of time was significant [*F* = 162.190, *p* < 0.001, partial η^2^ = 0.737, 95% CI (0.67, 0.79)]; The interaction between time and group was significant [*F* = 190.215, *p* < 0.001, partial η^2^ = 0.766, 95% CI (0.71, 0.81)]. Further simple effects analysis revealed that t3 in the experimental group was significantly higher than t1 and t2 (*p* < 0.000), but there was no significant difference between t1 and t2 (*p* = 0.719); In the control group, t2 was significantly higher than t1 (*p* = 0.022), but there were no significant differences between t3 and t1, or between t2 and t3 (*p* > 0.05).

The main effect of TPA in the experimental group was significant [*F* = 18.810, *p* < 0.001, partial η^2^ = 0.245, 95% CI (0.10, 0.39)]; The main effect of time was significant [*F* = 75.593, *p* < 0.001, partial η^2^ = 0.559, 95% CI (0.46, 0.64)]; The interaction between time and group was significant [*F* = 97.179, *p* < 0.001, partial η^2^ = 0.626, 95% CI (0.53, 0.70)]. Further simple effects analysis showed that t3 in the experimental group was significantly higher than t1 and t2 (*p* < 0.001), but there was no significant difference between t1 and t2 (*p* = 1.000); In the control group, t2 was significantly higher than t1 (*p* = 0.012), and t3 was significantly lower than t2 (*p* = 0.041), but there was no significant difference between t3 and t1 (*p* = 1.000).

## 4 Discussion

Research suggests that mastering motor skills does not necessarily motivate individuals to participate in PA, the key factor driving sustained engagement lies in the sense of fulfillment and enjoyment derived from PA (Whitehead, 2013). Appropriate physical activity not only reduces children’s risk of chronic diseases such as heart disease and diabetes but also improves quality of life and enhances wellbeing (World Health Organization, 2019; Britton et al., 2023). Additionally, MVPA typically involves higher levels of physical coordination and muscular engagement, which can promote the development of neural networks, bones, and muscles, thereby providing a physiological foundation for subsequent mastery of the FMS (Kara et al., 2019; Schmutz et al., 2020). This study implemented a 12-week structured physical education intervention program for preschool children aged 3–6 years. Using repeated measures analysis of variance, it examined the differential characteristics among different groups and age cohorts of preschoolers. After a 12-week experimental intervention, it was found that compared to conventional courses, the experimental curriculum demonstrated significantly greater effectiveness in reducing SB among preschoolers and enhancing MVPA and TPA among children aged 3–6 years. This validates the positive impact of LEG structured physical education programs on elevating PA in young children.

As shown in Table 4 regarding the SPA performance of 3–4-year-old children that the experimental group significantly outperformed the control group. Only VPA remained consistently higher than the control group, while SB, LPA, MPA, MVPA, and TPA all showed a decline from their peak levels. MVPA increased by 68.5% at 6 weeks compared to baseline (8.14 vs. 4.83 min), but this increase had dropped to 39.1% by 12 weeks. TPA also increased by 39% at 6 weeks (14.56 vs. 10.47 min), but by week 12, the increase had dropped to 7.6%. This indicates that physical activity interventions for children in this age group exhibit a phased characteristic of being “This indicates that physical activity int” This finding is consistent with the conclusion that 3- to 4-year-olds have short attention spans, strong independent minds, and difficulty sustaining high levels of MVPA and TPA (Fedewa et al., 2021), and it supports the view that interventions for young children require timely adjustments to activity content and format to maintain novelty (Bikila, 2024). Research indicates that 3–4-year-olds are still in the early stages of developing motor skills, attention, and the ability to understand rules. They are prone to frustration when faced with complex or sequential tasks, which is often accompanied by a decline in interest (MacDonald and McIntyre, 2019). Furthermore, the cognitive characteristics of their preoperational stage limit their ability to execute multi-step instructions. Therefore, in structured programs, it is not advisable to place excessive emphasis on meeting MVPA targets for 3–4-year-olds; instead, priority should be given to maintaining their motivation to participate and fostering positive emotional experiences.

Table 5 shows the SPA performance of 4–5-year-old children that the experimental group performed significantly better than the control group, and the intervention results were the most favorable among the three experimental groups. This was reflected in sustained improvements in SB, MVPA, and TPA, with SB time reduced to < 50%, and a decline in the peak levels of LPA and MPA. MVPA increased by 139.7% from week 0 to week 6 (12.78 vs. 5.33 min) and further increased by 30.1% by week 12. TPA increased by 84.5% at week 6 (21.14 vs. 11.46 min) and remained at a similar level by week 12. Observations suggest that 4–5-year-old children become more sensitive to relational needs and social interaction, and the social elements of the curriculum begin to play a motivating role. These findings support previous research indicating that 4–5-year-olds demonstrate significant improvements in gross and fine motor skills, enhanced cognitive abilities, and the ability to adapt to the constraints of structured curriculum tasks (Liu et al., 2022). Unlike 3–4-year-olds, who rely primarily on the novelty of activities, 4–5-year-olds place greater emphasis on peer relationships and a sense of task accomplishment. Social feedback may serve as a more sustained motivator than the activity’s inherent fun. The findings for this age group suggest the existence of an “optimal window of opportunity” for intervention, during which structured programs can simultaneously increase MVPA and reduce SB.

As shown in Table 6 regarding the SPA performance of 5–6-year-old children that the experimental group significantly outperformed the control group and exhibited a clear delayed effect. At 6 weeks, there was no significant difference compared to the control group. It was not until 12 weeks that the experimental group significantly outperformed the control group. Furthermore, the proportion of MVPA and TPA consistently remained below 50% of the class time. MVPA increased by only 13.1% from baseline to week 6 (3.98 vs. 3.52 min), but rose sharply by 251% at week 12 compared to week 6 (13.93 min). TPA also saw a significant increase at week 12 (a 140% increase compared to week 6). These findings are consistent with the view that MVPA during physical education classes decreases as class size increases (Skala et al., 2012; Kirkham-King et al., 2017). Furthermore, the trends observed in the 5–6-year-old group may also be influenced by a combination of factors, including class size (Pedersen et al., 2022), the allocation of teaching resources, curriculum content, academic task demands (Sigmund et al., 2009), and increased autonomy needs. This is inconsistent with the view that 5–6-year-old children have begun transitioning to the concrete operational stage, possess stronger information processing and attention maintenance abilities, and can benefit from high-cognitive-load tasks requiring reasoning and decision-making (Deng and Chen, 2023). Observations of courses reveal that this phenomenon is constrained by class size, course resources, and content. Consequently, there are additional factors influencing the impact of structured courses on this age group, which has clear methodological implications for the design of future interventions.

In this study, increases in young children’s MVPA and TPA levels were accompanied by a reduction in sedentary behavior, supporting the view that a reasonable allocation of time between skill practice and play in physical education classes can increase the proportion of MVPA (Zhou, 2020). This finding is consistent with the conclusion that increasing the duration of in-class interventions can enhance MVPA during physical education classes (Li and Wang, 2020). However, the findings of this study are inconsistent with the argument that MVPA levels are higher during free play than during organized activities (Maitland et al., 2013). This discrepancy may stem from differences in activity contexts: during free play, although young children have autonomy in their choices, they may spend a significant amount of time in low-intensity or sedentary states. In contrast, the LEG curriculum model uses structured segments to “distributively guide” activity intensity, thereby increasing overall MVPA levels and reducing SB.

We further analyzed the mechanism of action of the LEG curriculum model. The movement learning and practice components of this model provide opportunities for building a repertoire of movements and acquiring new skills, while the game activities create the time and space required for MVPA and demand a high level of physical coordination and muscular engagement. This aligns with the view that FMS can enhance young children’s sense of accomplishment and motivate sustained participation in MVPA (Bezerra et al., 2021; He et al., 2021; Graham et al., 2022). Furthermore, this study confirms the following consistent findings: structured curricula can reduce sedentary behavior in children and increase moderate-to-vigorous physical activity (Kara et al., 2019); organized activities elicit higher levels of physical engagement compared to free play (Palmer et al., 2017; Tortella et al., 2019; Pate et al., 2016) and structured physical education programs in early childhood settings are an effective means of increasing children’s MVPA (Hnatiuk et al., 2019). Thus, the LEG model facilitates a shift in young children’s physical activity from “inefficient time-wasting” to “high-efficiency exercise.”

Nevertheless, under this model, only children aged 4–5 met the recommended standard of at least 50% MVPA set by the United States (U.S. Department of Health and Human Services, 2010) and the United Kingdom (Association for Physical Education, 2015). Neither the 3–4-year-old nor the 5–6-year-old groups met this standard. These findings suggest that structured physical education programs are reliably associated with reduced SB, but there are significant differences in their effectiveness across age groups, and A single program model is unlikely to comprehensively optimize physical activity levels for young children of all ages.

Based on the above findings, structured curriculum design must explicitly incorporate age-differentiated strategies while balancing fun with skill development. For 3–4-year-olds, MVPA targets should be appropriately lowered, and diverse, short-duration, high-frequency activity transitions should be employed to maintain motivation to participate. For 4–5-year-olds, their social development needs can be fully leveraged by introducing peer collaboration and task feedback mechanisms to consolidate the benefits of MVPA. For 5–6-year-olds, attention must be paid to factors such as class size, competition over academic tasks, and curriculum content that may inhibit activity intensity.

However, the implementation of this model in early childhood education settings still faces several practical challenges. The structured design of the curriculum places high demands on teachers’ organizational skills, ability to demonstrate movements, and classroom management skills; larger class sizes may limit teachers’ ability to monitor individual exercise intensity, and the alignment of teaching resources with curriculum content may also affect children’s sustained engagement. If teachers lack appropriate training or teaching motivation, implementation effectiveness may be compromised. Implementation challenges are particularly pronounced in preschools with large class sizes or high teacher turnover. Although this model has demonstrated feasibility in reducing SB and increasing MVPA, its sustained health benefits require validation through longer-term, larger-scale studies.

## 5 Conclusion

The LEG structured curriculum model has been shown to be highly effective in reducing sedentary behavior and increasing physical activity levels among children aged 3–6, with the greatest effectiveness observed among 4–5-year-olds. This model reduces passive waiting and SB among young children during activities, creating more opportunities for them to engage in MVPA. However, intervention strategies still need to be further optimized to improve the sustainability and overall effectiveness of sedentary behavior and physical activity levels among children aged 3–4 and 5–6 years.

## 6 Limitations

This study has the following limitations. First, the study employed a single-center design, with all participants recruited from a single kindergarten located in an urban-rural fringe area in western China. Given the limited representativeness of the sample, caution is warranted when generalizing the study’s findings to other regions, cultural contexts, or types of kindergartens. Second, the intervention period lasted only 12 weeks. Although significant immediate effects were observed, the long-term efficacy and scalability of this model have not yet been confirmed. The study did not include long-term follow-up, and future research should extend the intervention period and conduct follow-up assessments. Third, the intervention was implemented by classroom teachers within the natural teaching environment, factors such as teachers’ teaching styles, rapport-building skills, and classroom management abilities may have potentially influenced the children’s activity levels. Although data were collected at regular assessment points, these confounding factors cannot be completely ruled out. Fourth, this study focused on changes in total physical activity volume and activity levels of varying intensities, but did not specifically examine the development of children’s FMS. Therefore, the comprehensive evaluation of the intervention’s effectiveness remains incomplete. Given these limitations, future research should focus on the following specific areas. First, further validating the generalizability of these findings in multicenter studies with diverse samples. Second, extending the follow-up period to assess long-term outcomes. Third, objectively monitoring the implementation and reliability of the intervention, and fourth, conducting an in-depth exploration of the developmental mechanisms underlying the differing responses to the intervention among children of various age groups.

## Data Availability

The original contributions presented in this study are included in the article/supplementary material, further inquiries can be directed to the corresponding author.
